# Editorial: Sex and gender differences in neurodegenerative diseases

**DOI:** 10.3389/fnins.2023.1175674

**Published:** 2023-03-16

**Authors:** Jessica Elaine Young, Minjie Wu, Holly C. Hunsberger

**Affiliations:** ^1^Laboratory Medicine and Pathology, Institute for Stem Cell and Regenerative Medicine, University of Washington, Seattle, WA, United States; ^2^Department of Psychiatry, University of Pittsburgh, Pittsburgh, PA, United States; ^3^Department of Neuroscience, Center for Neurodegenerative Diseases and Therapeutics, Rosalind Franklin University of Medicine and Science, North Chicago, IL, United States; ^4^Department of Foundational Sciences and Humanities, The Chicago Medical School, North Chicago, IL, United States

**Keywords:** sex differences, neurodegeneration, aging, inflammation, memory

Behavioral differences between men, women, boys, and girls, has been a topic of interest since the classical period of Plato and Aristotle. However, it wasn't until the early 1900s that scientists speculated the brain as the principle means by which males and females behave differently. In 1950, Frank Beach famously argued that genitalia were the critical variable that determined male vs. female behavior (Beach, 1974). This theory was overturned 9 years later by Dr. William Young who discovered that prenatal hormones were capable of sex reversing the behavior of females during adulthood (Phoenix et al., 1959). So why didn't this and other similar findings accelerate research into sex differences? Instead, what followed were narrow studies of reproductive behavior and physiology, leading to the myopic view that sex differences in the brain were limited to the anterior pituitary gland, courtship, copulation, and parenting (McCarthy et al., 2015). However, the seminal finding of Woolley and McEwen, that dendritic spine density on hippocampal neurons varied by 30% across the estrus cycle in female rats, led us out of the context of reproduction and into the idea that hormones can modulate neuronal plasticity (Woolley and McEwen, 1992).

Although our narrowed view had widened, these types of studies are difficult in adults and aging models because of the hormonal modulation in adulthood. Do we compare intact male and female rodents with estrus cycle fluctuations? Should both sexes be gonadectomized and hormone replaced? How costly will it be to include both sexes with multiple groups? This thinking led scientists to again limit their studies to mainly male rodents. The myth that females are variable and difficult to study has been disproved by several comprehensive studies noting that male data can be more variable (McCarthy, 2015; Shansky and Woolley, 2016). Therefore, in 2016 the NIH required that both male and female subjects be included in grant applications (SABV policy).

Sex and gender differences in disease prevalence and progression are relatively common (Dahodwala et al., 2018). For example, Parkinson's disease (PD) is more common in males, while AD is more prevalent in women (Beam et al., 2018). This editorial includes studies that aim to further the neurodegeneration field by including analysis by sex to better understand how disease progression, prescription medication, inflammation, and cognition differs in men vs. women. Unfortunately, we are limited to sex as a binary as these studies have yet to include non-binary and transgender people or are exclusively using rodent models. Future work is already beginning in this area as we continue to push our once narrow view of sex differences even further.

PD disproportionately affects men (2X more likely), but this sex difference is poorly understood as women have higher mortality and faster progression of the disease (Cerri et al., 2019). Risk factors for men include, genetic mutations, chronic stress, neurotoxic chemicals, traumatic brain injury, and diet (Hemmerle et al., 2012; Teschke et al., 2014; Rozani et al., 2018; Bakshi et al., 2019). Interestingly, the disease progression is also different in men vs. women as each display unique symptoms (Baba et al., 2005). In this regard, Oltra et al. revealed that PD males had greater motor and REM movement sleep behavior disorder symptoms compared to PD females as well as cortical thinning and smaller volumes in certain brain regions. Overall, PD males performed worse in global cognition, verbal recall, and processing speed. The mechanisms underlying these sex differences remain unknown and represent a unique area of study for future directions.

Although medications are lacking for these neurodegenerative disorders, the co-morbidities associated with aging, results in multiple prescription medications or polypharmacy for those suffering from PD or AD. Although it is clear that people with AD take significantly higher numbers of medication compared to age-matched controls (Clague et al., 2017), few studies have extended this line of research to examining how sex differences may further affect how medications impact risk of developing disease. There is evidence that therapeutics act in a sex-specific manner (Dodiya et al., 2019). Du et al. describes an in-depth analysis relating polypharmacy to health and cognitive performance. They discovered that the number of prescribed medications was associated with worse self-rated health and a faster decline in executive function, and that women took more, often inappropriate, prescription medications than men. There is a need for better medications and a better understanding of their side effects especially in an aging population. Future studies may want to turn their efforts toward treating overall inflammation which is observed in both PD and AD. Most neurodegenerative diseases are characterized by inflammation and therefore microglia activation (Hanamsagar and Bilbo, 2016). On the cellular level, Lynch discusses how sex differences in microglia might be a game changer in precision medicine. They note that microglia numbers begin to differ in the early postnatal period between male and female rats and sex-related difference continue throughout adulthood.

Neurodegeneration is not the only cause of cognitive decline, cognitive deficits are also observed after chronic exposure to hypobaric hypoxia, low oxygen pressure, which occurs at high altitudes. In recent years, many people have migrated to high-altitude areas as outdoor activities are becoming more common in part due to COVID-19. Cognitive deficits induced by exposure to hypobaric-hypoxia are maladaptive responses associated with oxidative stress and inflammation (Dheer et al., 2018). Previous reports have revealed that women are physiologically protected at high altitude until they reach menopause (Joseph et al., 2002). Zhu et al. notes that these studies suggest sex hormones make a difference and describes the role of ovarian hormones in rats exposed to hypobaric hypoxia. Male rats were more likely to develop hippocampal damage, neuroinflammation, and cognitive decline compared to females.

Overall, the research articles and the review that comprise this Research Topic expand our knowledge on sex differences in neurodegeneration and cognitive decline. This hope is to open new avenues for future sex-specific neurodegeneration studies with the goal of personalized therapeutics in mind.

## Author contributions

HH developed the outline of the editorial. Editing was completed by JY and MW. All authors contributed to the article and approved the submitted version.
